# COVID-19 in a Portuguese whole blood donor population

**DOI:** 10.1016/j.heliyon.2023.e20570

**Published:** 2023-10-02

**Authors:** Liliana Fonseca, Filipa Martins Pereira, Luís Moura, Arnaldo Brito, Filipe Lobo, Ana Palmira Amaral, Marina Costa

**Affiliations:** a Serviço de Sangue e Medicina Transfusional, Centro Hospitalar Tondela-Viseu, Portugal; b Serviço de Imunohemoterapia, Centro Hospitalar Universitário de Santo António, Portugal

**Keywords:** Whole blood donors, COVID-19, Prevalence, Blood group, Pandemic

## Abstract

Coronavirus disease 2019 (COVID-19) is an infectious disease caused by the severe acute respiratory syndrome coronavirus 2 (SARS-CoV-2), leading to pneumonia and acute respiratory distress syndrome. The COVID-19 pandemic had a major impact on the stock of blood banks worldwide. This study aims to assess the prevalence of COVID-19 in a population of whole blood donors and analyze the possible association between blood group and susceptibility to the disease and the impact of adopting preventive measures against SARS-CoV-2 infection. **Material and methods**: This retrospective study included all whole blood donors from a Portuguese hospital between July and September 2021. A self-assessment questionnaire was used to collect data on COVID-19 infection, vaccination, and preventive measures. Statistical analysis was performed using Chi-square and Mann-Whitney U tests. **Results**: The prevalence of COVID-19 in the donor population was 11.96% (n = 97), with only 2 cases of serious illness requiring hospitalization. No association was found between blood group and disease susceptibility. Older men were less likely to adopt preventive measures. The vaccination rate was high, with 84.26% of donors having received at least one dose of the vaccine. Seven donors declined COVID-19 vaccination. Preventive measures did not differ based on COVID-19 infection status or vaccination. **Discussion**: Although there was a higher frequency of COVID-19 in group A donors, the blood group was not associated with susceptibility to infection. The donor population consisted of young individuals without comorbidities, showing a COVID-19 prevalence like the general population and few severe cases. The high vaccination rate and adoption of preventive measures likely contributed to these findings.

## Highlights

• The blood donor and general population had similar COVID-19 prevalence.
• Our population adopts measures to prevent SARS-CoV-2 infection and adheres to vaccination.
• No association was found between blood group and disease susceptibility.

## 1 Introduction

In December 2019, a new coronavirus, SARS-CoV-2, was identified in Wuhan, China. This virus causes COVID-19, a respiratory illness with varying severity [1,2]. The infection rapidly spread, leading to a pandemic declaration by the World Health Organization in March 2020 [3].

The variability and susceptibility to SARS-CoV-2 infection soon led to the study of possible genetic and environmental risk factors for the disease.

Due to a previously reported association between polymorphism of the ABO blood and the susceptibility to, and outcomes of, several diseases and infections (including severe acute respiratory syndrome coronavirus 1, SARS-CoV-1) [4], there have been several studies evaluating this association in the pandemic context. The evidence demonstrates that blood groups A and B glycosyltransferases affect glycosylation in epithelial cells in the respiratory tract. Also, the interaction between the SARS-CoV-2 S protein and its membrane receptor angiotensin-converting enzyme 2 (ACE2) could be inhibited by anti-A blood group antibodies that are naturally present in blood groups O and B individuals. These biological mechanisms have been proposed to explain the association between blood groups and COVID-19 [4].

Currently, there are conflicting reports. An early study from China suggested an association between blood group A and increased susceptibility to the disease plus a reduced susceptibility in group O patients [5]. Subsequent studies from Italy and Spain [6] showed similar results, while North American studies showed discordant results [7,8]. One study reported that group O was associated with lower mortality [4]. These divergent results may occur for a range of reasons, including differences in the study population and insufficient ABO prevalence data to allow comparisons. Much has been said about this disease in the general population and the impact of the pandemic on blood donation [9]. We can find some seroprevalence studies in the literature; those kinds of studies make it possible to assess the immune response to SARS-CoV-2 infection or vaccination and obtain data on the relationship between infection and symptomatic disease. The SARS-CoV-2 seroprevalence for infection-induced antibodies in blood donors is estimated between 2.7% and 20.2% and 83.3% for combined infection- and vaccine-induced antibodies [[10], [11], [12]]. The differences observed could potentially be attributed to variances within the study population, sample size, infection rates, diagnostic tests used, study period, and lockdown procedures.

It was found early on during the pandemic that the risk of transmission of SARS‐CoV‐2 by transfusion was only theoretical [13]. The pandemic had a big impact on blood supplies in the form of decreased blood donations (ranging from 26 to 62.89% [14,15]) essentially due to donors’ health status, fear or lockdowns [14,16,17]. With this observational epidemiological study, conducted between July and September of 2021, we aimed to assess the prevalence of COVID-19 infection, vaccination rate against SARS-CoV-2, and blood group relationship with the disease in a population of young and healthy individuals, such as blood donors. This information has not been reported for Portugal, at the time of our study; and most of the studies we could find were performed among hospitalized patients [[18], [19], [20]]. As secondary outcomes, we intended to evaluate the impact of some comorbidities such as diabetes mellitus and smoking, and the adoption of measures to prevent and control the spread of SARS-CoV-2 infection on the susceptibility to the disease in our population of donors.

## 2 Methods

### 2.1 Study design

An observational study that includes blood donors from the Blood Bank of the Tondela-Viseu Hospital Center (CHTV), a central hospital, with 35 medical specialties, that serves a population of 300,000 inhabitants.

The study was conducted from July to September 2021 and included all potential blood donors enrolled in the CHTV Blood Bank who were invited to participate in the study. They were approached on arrival at the center when handing in the questionnaire for blood donation. Donors who agreed to participate in the study were given the questionnaire and informed consent for participation, both in paper format.

First-time donors who accepted to participate in the study but were deferred to donate blood were excluded from the study. They were excluded from blood group analysis and from disease susceptibility analysis. Donor and prospective donor sociodemographics data, as sex, age, body mass index, number of household members, job and literacy, were used to characterize the center's donor population.

Participation in the study was voluntary and all participants gave written consent. The study was approved by the Tondela-Viseu Hospital Center Ethics Committee and the Teaching, Innovation and Scientific Development Department (local reference number 18/June 05, 2021) and complies with National and Hospital regulations. The privacy and anonymity of participants were guaranteed, and the participation was not compensated (financially or otherwise) and was entirely voluntary, with no repercussions for those who did not want to participate. The participants who desired had the option to access the overall findings of the study.

### 2.2 Survey instrument

The study was conducted through the application of a self-assessment questionnaire that included questions about age, gender, educational qualifications, occupation, household, risk diseases for severe COVID-19, vaccination status, and history of COVID-19. Only diseases that are not exclusion criteria for total blood donation were evaluated. If the donor had COVID-19, questions were asked about presented symptoms, context of infection, use of health care, and need for hospitalization.

**Table 2:** Comparison between blood donors who had COVID-19 and donors who did not have COVID-19. Chi-square test was used for categorical variables and Mann-Whitney *U* test for continuous nonparametric variables. Statistical significance was defined at p < 0.05.

	Without COVID-19	With COVID-19	*p*-value
**Male gender, n (%)**	412 (58.2)	52 (53.6)	0.392
**Age in years, median [interquartile range]**	40.5 [17]	41 [13]	0.664
**Body mass index, median [interquartile range]**	25.61 [5.06]	26.75 [5.04]	0.120
**Diabetes mellitus, n (%)**	27 (4.0)	4 (4.3)	0.924
**Arterial hypertension, n (%)**	40 (6.0)	7 (7.4)	0.585
**Asthma, n (%)**	34 (5.1)	4 (4.3)	0.719
**COVID-19 vaccine**^a^**, n (%)**	609 (86.3)	68 (70.1)	<0.001
**Blood group**			
**A**	293 (42.0)	45 (48.9)	0.366
**B**	53 (7.6)	3 (3.3)	
**AB**	15 (2.2)	2 (2.2)	
**0**	336 (48.2)	42 (45.7)	
**Rh (D)**	575 (83.2)	72 (80.9)	0.585

a at least one dose.

**Table 3:** Analysis of association between prevention measures of SARS-CoV-2 infection transmission and various characteristics of the whole blood donor population. Chi-square test was used for categorical variables and Mann-Whitney *U* test for continuous nonparametric variables. Statistical significance was defined at p < 0.05.

	Never - occasionally	Wash hands			Alcohol gel				Mask In closed spaces				Mask on the street				2-m distance in closed spaces			
		Often	Always	Chi2 *p*	Never - occasionally	Often	Always	Chi2 *p*	Never - occasionally	Often	Always	Chi2 *p*	Never - occasionally	Often	Always	Chi2 *p*	Never - occasionally	Often	Always	Chi2 *p*
**Total**	8.6	40.9	49.0		8.1	33.6	56.9		1.0	5.0	92.6		11.9	47.4	39.4		7.5	52.3	38.8	
**Gender**				31.04 **<0.001**				34.35 **<0.001**				7.25 **0.027**				52.03 **<0.001**				21.92 **<0.001**
Male	11.4	46.9	41.7		10.3	40.6	49.0		1.5	6.5	92.0		18.3	49.5	32.3		10.5	55.5	34.0	
Female	5.0	34.1	60.8		5.3	25.1	69.6		0.3	3.2	96.5		3.5	46.0	50.4		3.6	49.7	46.7	
**Age**				11.82 **0.019**				28.30 **<0.001**				2.433 0.657				3.48 0.480				20.23 **<0.001**
18-29	7.3	50.3	42.4		6.8	36.7	56.5		0.6	5.1	94.4		12.4	51.4	36.2		15.3	52.0	32.8	
30-55	9.1	40.2	50.7		7.0	34.4	58.6		1.2	4.8	94.0		11.5	46.9	41.6		5.3	53.5	41.1	
>55	8.9	24.4	66.7		28.9	20.0	51.1		0.0	8.9	91.1		17.8	48.9	33.3		6.7	51.1	42.2	
**Body mass index**				9.91 **0.042**				5.47 0.242				7.01 0.135				4.18 0.382				2.49 0.647
<25	5.9	41.2	52.9		9.0	31.2	59.9		0	5.9	94.1		11.1	45.7	43.2		8.3	52.2	39.5	
25-30	10.4	43.8	45.8		7.7	38.9	53.4		1.2	4.7	94.1		14.2	49.0	36.8		6.5	55.7	37.8	
>30	13.1	35.2	51.6		8.2	30.3	61.5		2.5	4.9	92.6		9.8	49.2	41.0		8.2	48.4	43.4	
**Diabetes mellitus**				0.30 0.862				0.07 0.965				0.552 0.759				0.230 0.892				0.121 0.941
No	9.3	40.9	49.7		8.5	33.8	57.7		1.0	5.2	93.8		12.5	47.9	39.6		8.1	52.9	39.0	
Yes	6.5	41.9	51.6		9.7	32.3	58.1		0	3.2	96.8		9.7	48.4	41.9		6.5	54.8	38.7	
**Arterial hypertension**				1.31 0.520				5.29 0.071				1.690 0.430				0.263 0.877				0.473 0.789
No	9.4	41.3	49.3		8.1	34.9	57.0		0.8	5.3	93.8		12.3	47.8	39.9		7.8	53.2	38.9	
Yes	6.4	36.2	57.4		12.8	19.1	68.1		2.1	2.1	95.7		12.8	51.1	36.2		10.6	51.1	38.3	
**Asthma**				7.43 **0.024**				2.88 0.237				0.91 0.634				1.99 0.369				0.16 0.923
No	9.3	41.8	48.9		8.1	34.0	57.9		1	5.3	93.8		12.2	47.5	40.3		8.1	53.1	38.9	
Yes	7.9	21.1	71.1		15.8	28.9	55.3		0	2.6	97.4		15.8	55.3	28.9		7.9	50.0	42.1	
**Smoking**				4.47 0.346				1.189 0.880				7.33 0.120				11.22 **0.024**				12.89 **0.012**
Never	8	41.3	50.7		8.5	23.6	57.9		0.6	4.6	94.8		10.1	46.7	43.2		7	50.5	42.5	
Past smoker	9.1	39.8	51.1		6.8	34.1	59.1		1.1	5.7	93.2		15.3	51.1	33.5		6.3	61.4	32.4	
Smoker	13.1	46.4	40.5		9.5	36.9	53.6		3.6	6.0	90.5		17.9	51.2	31.0		14.3	52.4	33.3	
**Educational qualifications**				15.26 **0.004**				12.85 **0.012**				6.96 0.138				9.72 **0.046**				3.011 0.556
Basic school	12.8	29.5	57.7		15.4	24.4	60.3		2.6	7.7	89.7		16.7	43.6	39.7		9	51.3	39.7	
High school	9.0	46.3	44.8		7.9	37.6	54.5		1.3	4.3	94.4		13.2	50.2	36.5		6.6	55.2	38.1	
Academic degree	6.6	36.7	56.6		6.6	30.4	63.0		0	5.8	94.2		8.6	45.1	46.3		8.9	49.4	41.6	
**COVID-19 vaccine**				2.41 0.660				2.76 0.599				0.64 0.959				8.52 0.074				4.60 0.331
No	10.0	44.2	45.8		10.8	34.2	55.0		0.8	5.8	93.3		8.3	48.3	43.3		10.8	45.8	43.3	
Yes	8.6	41.2	50.2		7.7	34.3	58.1		1.0	5.0	93.9		12.4	48	39.4		7	54.4	38.6	
Do not want	0	28.6	71.4		14.3	14.3	71.4		0	0	100		42.9	42.9	14.3		14.3	42.9	42.9	
**COVID-19**				3.60 0.165				3.94 0.140				3.26 0.196				3.65 0.161				1.19 0.552
No	8.8	40.5	50.6		9.0	33.7	57.3		1.1	4.7	94.2		12.8	47.2	40		7.5	53.8	38.6	
Yes	8.2	50.5	41.2		3.1	37.1	59.8		0	8.2	91.8		6.2	52.6	41.2		7.2	48.5	44.3	

To assess the adoption of measures to prevent and control the spread of SARS-CoV-2 infection, five questions about protective behavior were asked. A 5-point Likert scale was offered ranging from never (1), rarely (2), occasionally (3), often (4) to always (5). The questions about protective measures were: (i) frequency of hand washing with soap and water; (ii) use of alcohol gel solutions; (iii) use of mask in closed spaces; (iv) wearing a mask on the street; and (v) distance of at least 2 m between people in meetings in closed spaces.

The weight and height of the donors were evaluated at the medical appointment for the selection of potential donors for blood donation.

### 2.3 Statistical analysis

The data collected through the application of the questionnaire were transcribed by the authors into a database. For the analysis of the relationship of adopting behaviors to prevent the spread of SARS-CoV-2, these were categorized into never-occasionally (1–3), a combination of the responses for never to occasionally, often (4) and always (5). The age was stratified into age groups (18–29, 30–55, >55 years). The Chi-square test was used for categorical variables. Continuous nonparametric variables were accessed by the Mann-Whitney *U* test. Statistical significance was defined at *p* < 0.05. Statistical analysis was performed using SPSS™ software version 27.

## 3 Results

During the period of July and September 2021, 974 potential blood donors came to the CHTV blood bank. Of these, 815 accepted and agreed to participate in the study. The participation rate was 83.7%. Four participants, despite having accepted to participate in the study, did not complete the questionnaire and were therefore excluded from the study. Sixteen candidates for first-time donation at CHTV, who agreed to participate in the study, were deferred for donation. As such, they were excluded from major analyses. As previously stated only sociodemographic data was used.

Table I presents the characterization of donors’ population who accepted to participate in the study. Most donors were men (men:female 468:343), and the median donor age was 41 years. Regarding smoking habits, past smoker refers to people who smoked in the past but did not smoke in the 6 months before participating in the study.

Regarding donors’ professions, most donors were armed forces or public security personnel (n = 158), and the second most frequent occupation was service and commerce professionals (n = 106). As for professions with a higher risk of infection by SARS-CoV-2, 43 donors were health professionals, 18 were home or daycare workers and 60 donors were workers in the field of education. Most donors have a medium to high degree of literacy, with 45.9% of donors having completed high school and 31.7% having an academic degree.

The prevalence of COVID-19 in CHTV blood donors was 11.9%. COVID-19 diagnosis was determined according to criteria established by the Portuguese General Health Department. Usually, the PCR test was preceded by a rapid antigen test. Of the 97 donors who had COVID-19, only 2 required hospitalization and none required intensive care. Seven donors with COVID-19 went to the emergency department because of COVID-19 symptoms, but most (64.9%) only used the health support telephone line (Linha Saúde 24). Most donors who had COVID-19 had symptoms (74.2%) and underwent a diagnostic test for such symptoms (41.2%). No significant association was found between the number of household members and SARS-CoV-2 infection (*p=*0.537), although most donors were infected by a household member (34.0%). The comparison between donors who had COVID-19 and those who did not have COVID-19 is shown in Table II. The majority of the donors without COVID-19 had blood group O (48.2%), slightly different from the ones who had COVID-19 where the predominant blood group was A (48.9%). Note that in the analysis of risk factors for COVID-19, only diseases and habits that do not constitute exclusion criteria for blood donation were considered. No statistically significant differences were found between the groups except for vaccination status. Donors who did not have COVID-19 had a higher rate of vaccination.

The relationship between gender, age group, literacy, comorbidities, vaccination status, history of COVID-19 and adoption of measures to prevent the spread of infection by SARS-CoV-2 is shown in.Table III Men were less likely to adopt preventive measures than women (washing hands, use of alcohol gel, wearing a mask on the street and keeping a 2-m distance in closed spaces, *p* < 0,001; wearing a mask in closed spaces, *p* = 0,027). Older people also adhere less to such measures (use of alcohol gel and keeping a 2-m distance in closed spaces, *p* < 0,001; washing hands, *p* = 0,019; wearing a mask in closed spaces, *p* = 0,657; wearing a mask on the street, *p* = 0,480). Compared to donors who do not have asthma, donors with asthma wash their hands more frequently with soap and water (48.9 vs. 71.1, *p* = 0.024). Smokers wear masks less often in the street (*p=*0.024) and do not maintain 2 m distance in closed spaces (*p=*0.012) compared to non-smokers. Less educated donors wash their hands more frequently with soap and water (*p=*0.004), and donors with higher levels of literacy tend to use alcohol gel solutions more frequently to disinfect their hands (*p=*0.012). Thirty percent of donors (n = 249) had been vaccinated in a professional setting and 60.8% (n = 493) of the donor population had a complete vaccination schedule. Seven donors said they did not want to be vaccinated against COVID-19. No difference in preventive measures was seen between different status of COVID-19 infection or vaccination.

## 4 Discussion

We obtained high levels of participation in the study and our population mirrors the distribution of ABO type in Portugal - the group A is the most frequent, followed by group O [21]. Therefore, we were able to study the association between blood group and susceptibility to COVID-19; as expected, and according to the most recent literature [7], we found no association. Nevertheless, that there is a tendency with higher frequency of COVID-19 in group A donors and higher frequency of group O among COVID-19 negative donors, and the absence of statistically significant differences may be due to the low sample power of the study. The prevalence of COVID-19 in our population (11.9%) is in line with studies of SARS-CoV-2 seroprevalence in a blood donor population [11,12]. On the last day of questionnaires collection, the total number of COVID-19 cases in Portugal was 1069279,^22^ for a population of 10344802 inhabitants according to the 2021 census [22] (COVID-19 infection rate was 10.3%). Some seroprevalence studies of SARS-CoV-2 in blood donors reported that donors who weren't vaccinated at the time presented positive antibody titers and they were unaware that they may been sick, probably due to mild or even asymptomatic infection. Other donors who weren't vaccinated and had increased antibody titers were aware of their past COVID, but waited until they were symptom-free to donate [10,11,23,24]. Our assessment of disease prevalence was based on the donor's self-report. As anticipated in a young population without comorbidities, the number of cases with serious illness was low. Only two whole blood donors required hospital care, and none required intensive care.

Some studies have reported cases of more severe disease in men, as well as sociodemographic differences [25]. Since only two donors had severe disease requiring hospitalization, the relationship between severity and sociodemographic differences was not evaluated. However, an exploratory analysis of the adoption of preventive measures for disease transmission and gender, age and literacy was carried out. Also, we don't have enough data to study the association between blood group and disease severity.

At the end of July 2021, the self-scheduling of vaccination against COVID-19 for individuals over 18 years of age in Portugal began. As such it was expected to find a high vaccination rate against COVID-19 in the blood donor population. In Portugal, all people over the age of 18 are eligible for blood donation. In early October 2021, more than 8 million Portuguese were fully vaccinated [26]. In our population, the vaccination rate was similar to that of the general population, with 83.95% of donors fully vaccinated or with at least one dose of the vaccine administered. The high vaccination rate found is in line with the efficiency of the Portuguese vaccination program and the good adherence of the Portuguese population to vaccination. Seven donors declared that they were not interested in being vaccinated, despite having no formal contraindication for vaccination. The study did not aim to analyze the reasons for not performing vaccination, but it is assumed that they are the same as those of the general population.

Although a difference in vaccination status was observed between donors who had contracted COVID-19 and those who had not, conclusions cannot be extracted. The study was carried out approximately 7 months after the start of vaccination against SARS-CoV-2 in Portugal. The population initially selected for vaccination were health professionals, the elderly, and the population at risk for health problems. The expansion of vaccination to the entire Portuguese population occurred around the beginning of our study (July 2021). As such, most donors infected with COVID-19 had not received the vaccine prior to contracting the virus. The time elapsed between infection and eligibility for vaccination at the time was 6 months, so part of the donors who had COVID-19 still would not have time (between illness and vaccine) to be vaccinated. The time interval between disease and eligibility for vaccination was not evaluated and conclusions about these differences cannot be made. Possibly these donors had not completed the time required for the administration of the vaccine after curing the disease. Note that none of the donors who had COVID-19 expressed the desire not to be vaccinated.

The high adherence to measures to control the spread of SARS-CoV-2 infection can be explained by the fact that the donor population is a young population (41 years vs 45.8 years median age in Portugal [27]), with some degree of academic differentiation and with a good sense of civility. It was surprising to verify that the obese population and those with diabetes mellitus, compared to the population without these comorbidities, did not show a greater tendency to adopt protective measures against the infection, since they are people with a greater probability of serious illness. However, the reduced number of people with diabetes included and the high adoption of measures to prevent and control the spread of SARS-CoV-2 infection in the entire population may explain the absence of differences between the two groups.

Our donor sample is younger, healthier and more instructed than the Portuguese population, and these differences make it difficult to extrapolate the data to the general population. Also, our study was conducted from July to September 2021, which was a few months following the worst wave of COVID-19 on our hospital (between December 2020 and February 2021). Further studies, temporally more distant from the epicenter of the pandemic and with a larger sample, must be performed.

We concluded that our data population, as expected for a population of benevolent blood donors, is young and healthy, not showing a high prevalence of complicated chronic diseases. This can be observed by the low rate of diabetes mellitus, high blood pressure and COVID-19 requiring hospitalization. As well, they adopt measures to prevent SARS-CoV-2 infection and adheres to vaccination against COVID-19. The blood group was not associated with susceptibility to infection.

## Data availability statement

The data that has been used is confidential.

## CRediT authorship contribution statement

**Liliana Fonseca:** Conceptualization, Data curation, Formal analysis, Investigation, Methodology, Project administration, Resources, Writing – original draft, Writing – review & editing. **Filipa Martins Pereira:** Conceptualization, Data curation, Formal analysis, Investigation, Methodology, Resources, Writing – original draft, Writing – review & editing. **Luís Moura:** Data curation, Resources, Writing – original draft, Writing – review & editing, Investigation. **Arnaldo Brito:** Data curation, Investigation, Resources, Writing – original draft, Writing – review & editing. **Filipe Lobo:** Data curation, Investigation, Resources, Writing – original draft, Writing – review & editing. **Ana Palmira Amaral:** Conceptualization, Data curation, Resources, Writing – original draft, Writing – review & editing. **Marina Costa:** Conceptualization, Data curation, Supervision, Writing – original draft, Writing – review & editing.

## Declaration of competing interest

The authors declare that they have no known competing financial interests or personal relationships that could have appeared to influence the work reported in this paper.

## Figures and Tables

**Table I:** Characterization of the population of donors who accepted to participate in the study.

Variable	Measurement
**Male gender, n (%)**	468 (57.7)
**Female gender, n (%)**	343 (42.3)
**Age in years, median [interquartile range]**	41 [15]
**Body mass index, median [interquartile range]**	25.76 [4.98]
**Diabetes mellitus, n (%)**	31 (3.82)
**Arterial hypertension, n (%)**	47 (5.8)
**Asthma, n (%)**	38 (4.7)
Smoking	
**Never smoked, n (%)**	549 (67.7)
**Past smoker, n (%)**^b^	176 (21.7)
**Smoker, n (%)**	84 (10.4)
Vaccines	
**Flu vaccine, n (%)**	78 (9.6)
**COVID-19 vaccine**^a^**, n (%)**	680 (83.8)
COVID-19 diagnosis**, n (%)**	97 (12.0)
**Hospital admission, n (%)**	2 (2.06)
Blood groups	
**A, n (%)**	340 (42.80)
**B, n (%)**	56 (7.0)
**AB, n (%)**	17 (2.1)
**0, n (%)**	382 (47.1)
**Rh (D), n (%)**	661 (83.1)

a at least one dose.

b not smoked in the last 6 months.

## Abbreviations

ACE2 –: Angiotensin-converting Enzyme 2
CHTV –: Tondela-Viseu Hospital Center
COVID-19 –: Coronavirus disease 2019
*p* -: Probability
SARS-CoV-1 –: Severe acute respiratory syndrome coronavirus 1
SARS-CoV-2 –: Severe acute respiratory syndrome coronavirus 2
