# Supplementary material for: COVID-19 in a Portuguese whole blood donor population
Source: Heliyon. 2023 Oct 2;9(11):e20570. doi: 10.1016/j.heliyon.2023.e20570 (PMC10651442; doi:10.1016/j.heliyon.2023.e20570)
Supplement: Multimedia component 1 [file mmc1.docx]

**Questionário do estudo:**

Etiqueta com número sequencial de dádiva

**COVID-19 numa População de Dadores de Sangue Total Portugueses**

1. Aceita participar neste estudo:

Sim (1) 🗆

Não (0) 🗆

1. Idade (anos): __________________________________________________________
2. Sexo:

Masculino (0) 🗆

Feminino (1) 🗆

1. Qual a sua profissão? ______________________________________________________________________
2. Qual o nível de ensino mais elevado que completou ou para o qual obteve equivalência? (Escolha apenas uma opção. Considere o seu último nível de escolaridade concluído com aproveitamento.)

Ensino básico 1º ciclo (equivale à 4ª classe) (1) 🗆

Ensino básico 2º ciclo (equivale ao 6º ano) (2) 🗆

Ensino básico 3º ciclo (equivale ao 9º ano) (3) 🗆

Ensino secundário ou pós-secundário (12º ano, cursos gerais, tecnológicos ou profissionais) (4) 🗆

Ensino superior (Licenciatura/Mestrado/Doutoramento) (5)🗆

Não responde (8)🗆

1. Por quantas pessoas é composto o seu agregado familiar? _______________________
2. Tem alguma das seguintes doenças:

7.1 Diabetes: Sim (1) 🗆 Não (0) 🗆 Não sabe (88) 🗆

7.2. Hipertensão: Sim (1) 🗆 Não (0) 🗆 Não sabe (88) 🗆

7.3. Asma: Sim (1) 🗆 Não (0) 🗆 Não sabe (88) 🗆

7.4. Nos últimos 12 meses esteve internado por alguma destas doenças?

Sim (1) 🗆 Não (0) 🗆

1. Relativamente ao consumo de tabaco:

Nunca fumei (0) 🗆

Ex-fumador (1) 🗆

Fumador (2) 🗆

1. No último ano fez a vacina da gripe?

Sim (1) 🗆

Não (0) 🗆

1. Alguma vez tomou a vacina anti-pneumocócica (contra a pneumonia)?

Sim (1) 🗆

Não (0) 🗆

1. Realizou algum teste SARS-CoV-2 (COVID-19)?

Sim, por sintomas (1) 🗆

Sim, por contacto com pessoa infetada (2) 🗆

Sim, por curiosidade (3) 🗆

Sim, pela entidade patronal (4) 🗆

Não (0) 🗆

Outra: _________________________________________________________________

1. Alguma vez foi diagnosticado COVID-19?
   1. Sim (1) 🗆 Data do teste positivo: _____________________________________

Não (0) 🗆

- 1. Se respondeu sim na pergunta anterior, responda às seguintes perguntas:
     1. Teve sintomas?

Sim (1) 🗆

Não (0) 🗆

- - 1. Data do início dos sintomas: (dd/mm/aaaa) _________________________
    2. Em que contexto foi infetado:

Agregado familiar (1) 🗆

Família que não o agregado familiar (2) 🗆

Amigos (3) 🗆

No trabalho (4) 🗆

Não sabe/ Não responde (88) 🗆

- - 1. Recorreu aos Cuidados de Saúde pelo COVID-19?

SIM – Serviço de urgência (1) 🗆

SIM – Médico de Família (2) 🗆

SIM – Linha Saúde 24 (3) 🗆

Não (0) 🗆

- - 1. Teve de ser internado pela doença COVID-19?

Sim (1) 🗆

Não (0) 🗆

- - 1. Esteve internado numa Unidade de cuidados intensivos devido à doença COVID-19?

Sim (1) 🗆

Não (0) 🗆

- - 1. No decorrer da doença teve algum evento trombótico como trombose venosa profunda ou embolia pulmonar?

Sim (1) 🗆 Qual? _______________________________________________

Não (0) 🗆

1. Durante a pandemia em algum momento teve de ficar em quarentena?

Sim (1) 🗆

Não (0) 🗆

- 1. Se sim, quantas vezes? _____________________________________________

1. Relativamente às medidas de prevenção e controlo da propagação da infeção por SARS-CoV-2 (COVID-19), indique em que medida as aplica no seu dia a dia:
   1. Lavagem frequente das mãos com água e sabão:

| Nunca | Raramente | Ocasionalmente | Quase sempre | Sempre |
| --- | --- | --- | --- | --- |
|  |  |  |  |  |

- 1. Uso de soluções à base de álcool gel:

| Nunca | Raramente | Ocasionalmente | Quase sempre | Sempre |
| --- | --- | --- | --- | --- |
|  |  |  |  |  |

- 1. Uso de máscara em estabelecimentos fechados:

| Nunca | Raramente | Ocasionalmente | Quase sempre | Sempre |
| --- | --- | --- | --- | --- |
|  |  |  |  |  |

- 1. Uso de máscara na via pública (quando circula a pé na rua):

| Nunca | Raramente | Ocasionalmente | Quase sempre | Sempre |
| --- | --- | --- | --- | --- |
|  |  |  |  |  |

- 1. Distanciamento de pelo menos 2 metros de outras pessoas em convívios em espaços fechados:

| Nunca | Raramente | Ocasionalmente | Quase sempre | Sempre |
| --- | --- | --- | --- | --- |
|  |  |  |  |  |

1. Já foi vacinado para COVID-19?

Sim (1) 🗆

Não (0) 🗆

Não quero ser vacinado (2) 🗆

- 1. Se respondeu sim na pergunta anterior:
     1. Em que contexto foi chamado para a vacinação:

Pela idade (1) 🗆

Pela profissão (2) 🗆

Outra: ______________________________________________________

- - 1. Quantas doses da vacina fez?

1ª dose (1) 🗆

1ª e 2ª dose (2) 🗆

Data da última dose

1. Caso ainda não tenha realizado a vacina, tem alguma contraindicação para a mesma?

Sim (1) 🗆

Não (0) 🗆
