# Supplementary material for: COVID-19 in a Portuguese whole blood donor population
Source: Heliyon. 2023 Oct 2;9(11):e20570. doi: 10.1016/j.heliyon.2023.e20570 (PMC10651442; doi:10.1016/j.heliyon.2023.e20570)
Supplement: Multimedia component 2 [file mmc2.docx]

INFORMAÇÃO AO PARTICIPANTE NO ESTUDO

**COVID-19 numa População de Dadores de Sangue Total Portugueses**

**Qual é o objetivo deste estudo?**

Estudar a possível associação entre grupo sanguíneo e suscetibilidade para infeção por SARS-CoV-2 numa população de indivíduos jovens e saudáveis, como é o caso dos dadores de sangue, assim como caracterizar a população de dadores quanto à infeção por SARS-CoV-2.

**Em que consiste a minha participação no estudo?**

Preencher um breve questionário acerca de vários aspetos relacionados com a sua saúde, incluindo informação sobre infeção prévia por SARS-CoV-2 e estado de vacinação contra SARS-CoV-2.

**Porque é importante eu participar?**

A sua participação é importante para contribuir para o conhecimento sobre possível associação entre grupo sanguíneo e suscetibilidade para infeção por SARS-CoV-2.

**Como são selecionados os participantes?**

Todos os dadores de sangue do Centro Hospitalar Tondela-Viseu entre julho e setembro de 2021 serão convidados a participar.

**Que benefício terei em participar?**

A sua participação ajudará a melhorar o conhecimento sobre o possível associação entre grupo sanguíneo e suscetibilidade para infeção por SARS-CoV-2 e qual o estado de imunização contra o SARS-CoV-2.

**Existem riscos associados à minha participação?**

Não existem riscos associados à sua participação no estudo. Realizará a sua dádiva de sangue como habitualmente, apenas terá de preencher um breve questionário.

**Quem terá acesso à minha informação?**

A informação colhida será codificada e apenas os profissionais do serviço de sangue terão acesso à informação não codificada. O anonimato do seu questionário é garantido através da atribuição de um número sequencial, não contendo dados diretos de identificação. Todos os profissionais envolvidos na recolha de dados, tratamento de amostras e análise de dados são sujeitos a sigilo profissional.

Durante a análise de dados, todos os dados estão anonimizados não permitindo identificar a sua identidade. Todos os resultados publicados em relatórios ou artigos científicos não permitirão a identificação individual de nenhum participante.

**Quais são os meus direitos se participar?**

A sua participação neste estudo é inteiramente voluntária. Pode recusar colaborar neste estudo sem ter que apresentar qualquer justificação e sem qualquer consequência.

DECLARAÇÃO DE CONSENTIMENTO

**COVID-19 numa População de Dadores de Sangue Total Portugueses**

Fui informado(a) sobre os objetivos gerais do estudo, e compreendi com clareza o que me é pedido como participante, nomeadamente:

1. Os dados a fornecer serão estritamente confidenciais. Assim que os procedimentos do estudo o permitam, esses dados e resultados serão tornados anónimos, isto é, deixarão de poder ser relacionados com a minha identificação;

2. Todos os investigadores que utilizem esses dados estarão obrigados a segredo profissional;

3. Os resultados do estudo que venham a ser tornados públicos nunca incluirão o meu nome ou qualquer elemento que permita identificar-me.

Nestas condições, declaro que aceito participar no estudo.

Data: _____ / __________________ / 2021

***Nome do participante: ________________________________________________***

***Médico Setor da Dádiva: _______________________________________________***
