# Supplementary material for: COVID-19 in a Portuguese whole blood donor population
Source: Heliyon. 2023 Oct 2;9(11):e20570. doi: 10.1016/j.heliyon.2023.e20570 (PMC10651442; doi:10.1016/j.heliyon.2023.e20570)
Supplement: Multimedia component 3 [file mmc3.docx]

**Questionnaire:**

**COVID-19 in a Portuguese Whole Blood Donor Population**

1. Do you accept to participate in this study?

Yes (1) 🗆

No (0) 🗆

1. Age (years): __________________________________________________________
2. Sex:

Male (0) 🗆

Female (1) 🗆

1. What is your job? ______________________________________________________________________
2. What is the highest level of education you completed or for which you obtained equivalence? (Choose just one option. Consider your last level of education successfully completed.)

Primary school – 1^st^ cycle (equivalent to 4^th^ grade) (1) 🗆

Middle school – 2^nd^ cycle (equivalent to 6^th^ grade) (2) 🗆

Middle school – 3^rd^ cycle (equivalent to 9^th^ grade) (3) 🗆

High school or equivalent (12^th^ grade, general courses, technological or professional courses) (4) 🗆

College Education (Bachelor/Master/PhD) (5)🗆

Do not reply (8)🗆

1. How many people constitute your household (including yourself)? _______________________
2. Do you have any of the following diseases?

7.1 Diabetes mellitus: Yes (1) 🗆 No (0) 🗆 Do not know (88) 🗆

7.2. Arterial hypertension: Yes (1) 🗆 No (0) 🗆 Do not know (88) 🗆

7.3. Asthma: Yes (1) 🗆 No (0) 🗆 Do not know (88) 🗆

7.4. In the last 12 months have you been hospitalized for any of these diseases?

Yes (1) 🗆 No (0) 🗆

1. Smoking habits:

Never smoked (0) 🗆

Past smoker (1) 🗆

Smoker (2) 🗆

1. Did you get the flu vaccine last year?

Yes (1) 🗆

No (0) 🗆

1. Have you ever had the pneumococcal vaccine (against pneumonia)?

Yes (1) 🗆

No (0) 🗆

1. Have you performed a SARS-CoV-2 test? (COVID-19)?

Yes, due to symptoms (1) 🗆

Yes, due to contact with an infected person (2) 🗆

Yes, out of curiosity (3) 🗆

Yes, required by the employer (4) 🗆

No (0) 🗆

Other: _________________________________________________________________

1. Have you ever been diagnosed with COVID-19?
   1. Yes (1) 🗆 Positive test date: _____________________________________

No (0) 🗆

- 1. If you answered Yes to the previous question, answer the following questions:
     1. Did you have symptoms?

Yes (1) 🗆

No (0) 🗆

- - 1. Date of onset of symptoms: (dd/mm/yyyy) _________________________
    2. In what social context were you infected?

Household (1) 🗆

Family other than the household (2) 🗆

Friends (3) 🗆

At work (4) 🗆

Do not know/ Do not reply (88) 🗆

- - 1. Did you use Healthcare Services due to COVID-19?

Yes – Emergency Department (1) 🗆

Yes – Family Doctor (2) 🗆

Yes – Linha Saúde 24 (3) 🗆

No (0) 🗆

- - 1. Did you have to be hospitalized for COVID-19?

Yes (1) 🗆

No (0) 🗆

- - 1. Have you been admitted to an intensive care unit due to COVID-19?

Yes (1) 🗆

No (0) 🗆

- - 1. During the course of the disease, did you have any thrombotic events such as deep vein thrombosis or pulmonary embolism?

Yes (1) 🗆 Which one? __________________________________________

No (0) 🗆

1. During the pandemic, did you ever have to be quarantined?

Yes (1) 🗆

No (0) 🗆

- 1. If Yes, how many times? ____________________________________________

1. Regarding measures to prevent and control the spread of SARS-CoV-2 (COVID-19) infection, indicate to what extent you apply them in your daily life:
   1. Washing hands with soap and water:

| Never | Rarely | Occasionally | Often | Always |
| --- | --- | --- | --- | --- |
|  |  |  |  |  |

- 1. Using alcohol gel-based solutions:

| Never | Rarely | Occasionally | Often | Always |
| --- | --- | --- | --- | --- |
|  |  |  |  |  |

- 1. Using mask in closed spaces:

| Never | Rarely | Occasionally | Often | Always |
| --- | --- | --- | --- | --- |
|  |  |  |  |  |

- 1. Wearing a mask on the street (when walking):

| Never | Rarely | Occasionally | Often | Always |
| --- | --- | --- | --- | --- |
|  |  |  |  |  |

- 1. Distancing at least 2 meters from other people when gathering in closed spaces:

| Never | Rarely | Occasionally | Often | Always |
| --- | --- | --- | --- | --- |
|  |  |  |  |  |

1. Have already been vaccinated for COVID-19?

Yes (1) 🗆

No (0) 🗆

I don't want to (2) 🗆

- 1. If you answered Yes to the previous question:
     1. In what context were you called for vaccination?

Due to age (1) 🗆

Due to profession (2) 🗆

Other: ______________________________________________________

- - 1. How many doses of the vaccine did you take?

1^st^ dose (1) 🗆

1^st^ and 2^nd^ dose (2) 🗆

Last dose date (dd/mm/yyyy): _____________________

1. If you haven't been vaccinated yet, do you have any contraindications for it?

Yes (1) 🗆

No (0) 🗆
